# ID 340 - Sustentabilidade e Custo para Aquisição de Inseticidas Utilizados no Programa de Vigilância, Prevenção e Controle do *Aedes aegypti* no Brasil (2002 A 2023)

**DOI:** 10.5327/2237-9622.2025.v34s1.340

**Published:** 2025-11-25

**Authors:** Tatiane Fernandes Portal de Lima Alves da Silva, Kauara Brito Campos, Walter Maia Ramalho, Henry Maia Peixoto

**Keywords:** dengue, *Aedes aegypti*, vigilância em saúde pública, custos de programa

## Abstract

**Introdução:**

Em 2024, nas Américas, foram registrados 11.710.162 casos de dengue, com 6.651 mortes, sendo 9.569.467 casos prováveis e 5.303 mortes no Brasil. O controle da infestação por gera custos significativos, especialmente durante surtos. Este estudo analisou os inseticidas utilizados no controle do no Brasil, correlacionando a incidência de dengue e os custos do Ministério da Saúde (MS) entre 2002 e 2023.

**Método:**

Trata-se de uma análise de custos baseada na aquisição de inseticidas pelo programa nacional de vigilância, prevenção e controle do do MS, bem como adquiridos por outros programas com efeito sobre esse vetor. Os dados foram obtidos do Sistema de Informações de Insumos Estratégicos (Sies) do MS, por meio da Lei de Acesso à Informação. As taxas de incidência foram calculadas para o Brasil a partir dos dados disponibilizados no Tabnet pelo Departamento de Informação e Informática do Sistema Único de Saúde (DataSUS). Foram considerados os casos prováveis autóctones, sendo excluídos os casos ignorados/branco, descartados e inconclusivos. A análise exploratória dos dados, incluindo a correlação de Spearman, foi realizada na plataforma Google Colaboratory, utilizando as bibliotecas Pandas e Seaborn do Python 3.10.

**Resultados:**

Os custos com a aquisição de inseticidas para as ações de controle do no Sistema Único de Saúde (SUS) têm aumentado ao longo dos anos, variando de R$ 8,9 milhões em 2003 para R$ 138,4 milhões em 2023. Em 2002, a aquisição de inseticidas para o representava 99% dos custos totais do MS. Observou-se, em 2023, que o custo de aquisição de inseticidas para o controle do em relação ao custo total destinado às compras de inseticidas do MS (incluindo os demais vetores) reduziu para 82%. Os maiores custos na série aconteceram em 2021 (R$ 328,1 milhões), quando o MS substituiu o larvicida piriproxifeno pelo biolarvicida espinosade DT 7,48%, e em 2022 (R$ 239,7 milhões), quando passou a distribuir a (Bti) para alguns municípios do País. As substituições por produtos biológicos e formulações compostas, de custo muito mais elevado, ocorreram a partir de 2020, após detecção de resistência de populações do mosquito aos inseticidas em uso até então.

**Conclusão:**

A hipótese inicial do estudo sugeria uma relação linear significativa entre a taxa de incidência e o custo anual de aquisição de inseticidas para o controle do ou outros inseticidas com efeito nesse vetor. Os resultados da correlação de Spearman indicam que, embora haja tendência de aumento em ambas as variáveis ao longo do tempo, a correlação foi fraca (r=0,22) e sem significância estatística (valor p=0,31). Esses achados sinalizam para possíveis explicações que incluem a presença de fatores intervenientes, tais como condições climáticas favoráveis ao vetor, resistência a inseticidas e manejo de produtos para controle químico. A interseção entre mudanças climáticas e a disseminação de doenças como a dengue exige uma abordagem integrada e atualizada. A revisão das diretrizes nacionais que regulamentam o uso de inseticidas no SUS, o fortalecimento da vacinação e investimentos em mobilização social, saneamento e pesquisa de avaliação econômica completa com foco nas novas tecnologias de vigilância, prevenção e controle do são cruciais para enfrentar esse desafio de forma sustentável.

